# Changes in food preferences and ingestive behaviors after glucagon-like peptide-1 analog treatment: techniques and opportunities

**DOI:** 10.1038/s41366-024-01500-y

**Published:** 2024-03-07

**Authors:** Sahana Bettadapura, Katherine Dowling, Kelli Jablon, Ahmed W. Al-Humadi, Carel W. le Roux

**Affiliations:** 1 Department of Neurosurgery, University of Arkansas for Medical Sciences, Little Rock, AR USA; 2 Brown University School of Public Health, Providence, RI USA; 3 Renaissance School of Medicine, Stonybrook University, Stonybrook, NY USA; 4 Diabetes Complications Research Centre, University College Dublin, Belfield, Ireland; 5 Diabetes Research Centre, Ulster University, Belfast, UK

**Keywords:** Obesity, Gastrointestinal hormones, Weight management

## Abstract

**Background:**

Glucagon-like peptide-1 (GLP-1) analogs are approved for the treatment of obesity in adults and adolescents. Reports have emerged that the weight loss effect of these medications may be related to changes in food preferences and ingestive behaviors following the treatment. Understanding the mechanisms which impact ingestive behavior could expand opportunities to develop more refined and personalized treatment options for obesity.

**Methods:**

Recent studies investigating the relationship between GLP-1 analogs and ingestive behaviors were retrieved from PubMed using the search terms: “obesity,” “food preference,” “taste,” “ingestive behavior,” “weight loss medication,” “anti-obesity medication,” “GLP-1 analog,” “tirzepatide,” “liraglutide,” “semaglutide.” Measurement tools were studied to compare variables used to assess food intake behavior. The main outcomes from each study were analyzed to evaluate the current standing and future directions of appetitive, ingestive, and consummatory behaviors and their association with GLP-1 analogs.

**Results:**

Thus far, studies have primarily explored the weight loss phase and report decreased short-term appetite and food intake upon treatment. However, research during the weight maintenance phase and objective measurements of food intake are notably sparse. Additionally, verbal reports have been primarily used to examine food intake, which can be susceptible to subjectivity.

**Conclusions:**

Elucidating the relationship between GLP-1 analogs and ingestive behavior could reveal additional parameters which contribute to their anti-obesity effects. To better understand these mechanisms, it is imperative to consider objective measurements of food intake in future studies. Several measurement tools have been adapted to measure variables of food behavior in humans, and each must be carefully considered with their strengths and limitations to develop optimal investigations.

## Introduction

Obesity is currently managed through three primary methods: lifestyle changes (e.g., diet modifications), surgical intervention (e.g., gastric bypass, gastric banding, sleeve gastrectomy), and pharmacotherapy using anti-obesity medications. Pharmacological intervention is increasingly utilized due to promising weight loss effects, minimal invasiveness, and sustainability. There are currently seven medications approved for the treatment of obesity: orlistat, naltrexone-bupropion, phentermine-topiramate, setmelanotide, tirzepatide, liraglutide, and semaglutide. In recent years, the latter two, belonging to a class of medications known as glucagon-like-peptide 1 (GLP-1) analogs, are prescribed more frequently. Weight loss efficacy while resulting in relatively few adverse events are major attractions to their use. This prompts further investigation and clarification of the mechanisms promoting weight loss, including their effect on ingestive behavior and food preference. Leveraging this understanding could help refine anti-obesity treatment to benefit clinicians and patients. Therefore, this review focuses on semaglutide and liraglutide and aims to explore current discoveries of their effect on ingestive behavior. We also evaluate the tools and techniques used to study these dimensions, and recommend future directions for further research.

Endogenous GLP-1 is synthesized by L-cells of the distal small intestine and is released in response to carbohydrate consumption. GLP-1 receptors are expressed in the gastrointestinal tract, pancreas, hypothalamus, heart, kidney, and lungs [1, 2]. Acute GLP-1 infusions promote insulin secretion, inhibit glucagon secretion, delay gastric emptying, and promote satiety [3, 4].

Food-intake is a result of behaviors and not a behavior in its own right. Thus, understanding the neurobiological mechanisms that alter behaviors to determine food intake is fundamental to understanding the association between appetitive behaviors and consummatory behaviors. Additionally, it is important to consider food preference as a separate variable which describes a subject’s food choice based on their personal enjoyment and satisfaction. These behaviors can be studied in both humans and animals and can yield important insights that assist clinicians in managing patient expectations when using anti-obesity treatments.

Verbal reports as a means of gathering information on ingestive behaviors are often used [1]; however, verbal report comes with an array of challenges that reduce the validity and reliability of the data obtained. For instance, verbal reporting relies heavily on the participant’s ability to accurately remember the parameters being studied [5]. Additionally, participants could be influenced by a social desirability bias and respond by altering their reports to reflect behaviors that are deemed more socially acceptable [6]. Verbal reports are also subjective by nature and thus participants might perceive and express their experiences differently.

Measuring food-intake directly, without relying on verbal reports, is an objective means of collecting ingestive behavior data, but there are practical challenges. Precise measurements may require the use of expensive devices that are complex to use, while also making long-term data collection particularly difficult. This could add significant cost and complexity to a study. Additionally, the act of directly measuring food intake can be invasive by nature, making subjects feel uncomfortable and subsequently risking the introduction of reactivity bias [7]. Direct observation of ingestive behaviors may also introduce social desirability bias by unintentionally causing participants to alter their normal activity in a conscious or subconscious effort to align with social norms regarding food intake [7].

Overall, while each tool has their associated strength, results obtained from techniques such as verbal reports run a greater risk of subjectivity due to individual interpretation, while direct measurements of food intake are more likely to provide objective and quantifiable data [5, 7].

Due to the prevalence of post-operative weight regain, anti-obesity medications are often prescribed to supplement the effects of bariatric surgery [8]. The link between anti-obesity medications and bariatric surgery, particularly gastric bypass, is strengthened by evidence of gastric bypass increasing gut hormone secretion and activity. Notably, GLP-1 and peptide YY secretion is accelerated upon gastric bypass, both of which are known to play key roles in appetite regulation and hunger reduction [9, 10]. This connection supports the feasibility of using investigations of ingestive behavior following gastric bypass surgery as models for similar studies upon anti-obesity medication treatment.

Previous studies investigating ingestive behaviors after gastric bypass surgery have provided valuable insights into procedure-associated weight loss [5, 11, 12]. While the significantly decreased volume of food consumed by patients leads to decreased caloric intake, gastric bypass surgery also causes notable changes to gut hormone levels [5]. Plasma levels of gut hormones start rising within 15 min of starting a meal and typically peak around 30–90 min. Satiation (resulting in a meal being stopped) occurs much sooner than the peak of the plasma levels. Satiety (resulting in a delay of the onset of the next meal) often continues even after the plasma levels have started to return to baseline. The duration of satiety is associated with the height of the peak of the gut hormones and the duration before the gut hormones return to baseline [13]. These gut hormone changes contribute to reduced hunger and appetitive behavior [5]. Gastric bypass in rodents results in weight loss, reduced food-intake, reduction in preference for sucrose and intralipid during a two-bottle preference test, reduced consumption of fat, with a modest increase in protein intake [11, 12]. Whilst in humans, gastric bypass results in weight loss, reduced food intake, and reduction in rate of eating, but no change in food selection or frequency of eating [14]. However, conclusions regarding changes in food selection after gastric bypass continue to be evasive, with various studies reporting differing results depending on the technique employed [5]. Furthermore, during the weight loss phase in humans, the progressive ratio task (PRT), an operant measurement which requires participants to complete a varying amount of tasks to receive a reward, shows a reduction in appetitive behavior, whereas in rodents in the weight loss maintenance phase, there are no changes in appetitive behavior [15, 16]. In rodents, taste reactivity as a measure of consummatory behavior shows no change in the palatability of food at the start of the meal, but an increase in aversive behavior is observed when the meal has ended [17].

Appetitive behavior describes the effort the subject is willing to undertake to reach a reward such as food [18]. These responses can include food-seeking behaviors which lead to food consumption and can result in food foraging or food hoarding [18]. Food hoarding as an appetitive behavior has been extensively studied in non-human primates with laboratory animals exhibiting increased hoarding following food deprivation [19]. However, food intake does not necessarily increase, demonstrating a delineation between appetitive and ingestive behavior [19]. When translating this phenomenon to humans, studies of food hoarding and other appetitive behaviors as a result of fasting or food deprivation are relatively limited [10].

Appetitive behavior can be influenced by neuroendocrine mechanisms [18]. Siberian hamsters which were administered neuropeptide Y (NPY) injections to the hypothalamic paraventricular nucleus and perifornical area displayed marked increases in food hoarding, highlighting it as a key regulator of appetitive behavior [20]. Ghrelin, a hormone stimulating gastric emptying and appetite, increases upon fasting in animals such as hamsters [21]. Ghrelin injections in hamsters stimulate long-term food hoarding, highlighting a major neurochemical factor in appetitive behavior [22]. Moreover, studies conducted on gerbils have shown food hoarding to increase activation of cells containing tyrosine hydroxylase, which catalyzes rate-limited dopamine synthesis, in subcortical regions of the brain [18]. This suggests an element of reward and reinforcement driving appetitive behavior [18].

The PRT is a direct measure of appetitive behavior [15]. This technique is widely used to investigate the hedonic value reinforcers in animal models which can answer the question of how hard a subject is willing to work for a given reinforcer [23–26]. The method was built on previous studies that used PRTs in humans [27–33]. One of the key merits of the task is that the assessment is based on the actual behavior of the subject and is not burdened by the interpretive limitations associated with scaling procedures using verbal reports. The PRT uses simple computer software and requires participants to consume the reinforcer during the task rather than at the end. Thus, appetitive responsiveness is determined directly by the orosensory properties (e.g., taste) of the reward and is independent of the association between a stimulus such as a token, money, or images with the reward. The completion of the task is not dependent on the technological or intellectual skills of the participant. To minimize post-ingestive effects, reinforcers are of minimal volume and calories so that the ingestion of a food reward may not lead to premature satiety and interfere with the oral-based evaluation. This property makes PRT beneficial for studying changes in appetitive responsiveness in patients [5]. Participants are briefed about each experiment by an investigator who is not present during the task to minimize bias in responses. These methodologic features differentiate this paradigm from others used in humans in previous studies [10–16].

Consummatory behaviors occur when there is direct interaction between the taste buds and food and are thought to be highly regulated by neuroendocrine mechanisms [18]. Ghrelin, in addition to proteins of the hypothalamic-pituitary-adrenal and hypothalamic-pituitary-gonadal axes, has been shown to impact consummatory behavior in laboratory animals [18]. Quantitative studies on consummatory behavior following anti-obesity medication are relatively sparse, requiring further exploration.

When assessing consummatory behavior, it is important to consider the types of food that patients who undergo anti-obesity treatment choose to consume. When evaluating mechanisms of weight loss, sweet and fatty food consumption trends can be particularly informative. Previous works indicate that sugar and fat preferences decreased in rats that underwent gastric bypass surgery [16, 34]. These results have not been replicated in humans undergoing gastric bypass surgery [35]. Furthermore, food selection in the context of anti-obesity medications has not been studied extensively. Therefore, this study aims to shed light on current research regarding the effect of GLP-1 analog pharmacotherapy on food intake and behaviors, their limitations and opportunities to expand, and methods used to assess these dimensions.

## Methods

This article reviews studies which shed light on the relationship between GLP-1 analogs and ingestive behaviors, while reviewing measurement tools utilized to obtain results. The following search terms were applied in PubMed to locate relevant articles: obesity, food preference, taste, ingestive behavior, weight loss medication, anti-obesity medication, GLP-1 analog, tirzepatide, liraglutide, semaglutide. Additional filters were applied to obtain studies inclusive of humans and/or rodent subjects. Age and language criteria were not accounted for as search criteria. Studies were included if they were published within the last 25 years to ensure the research is up to date but still ensure there were sufficient resources, given that it is an understudied field. Eligibility was also restricted to articles that discussed GLP-1 analogs and specifically analyzed taste preference along with ingestive behavior following treatment. For the scope of this review, articles which investigated GLP-1 analogs as treatments for obesity were primarily extracted, although their usage is also utilized in the treatment of type 2 diabetes. Titles and abstracts were reviewed for relevance and to determine if the study fit within the eligibility criteria. Articles were ensured to be current and relevant to the timeframe in which the study was conducted (July 2023 – October 2023). Selected articles were downloaded to and managed from Mendeley, which was also used for citation management. The following data were extracted from the studies: study type, year of publication, location of study, study population, methods, and key results. Further terms were then applied, including “universal eating monitor,” “drinkometer,” “visual analog scale,” “Leeds food preference task,” and “control of eating questionnaire” in conjunction with the aforementioned search terms to examine the various methods for assessing ingestive behavior. Relevant studies with both human and animal subjects were extracted and applied. The main outcomes analyzed from each study are the impact of GLP-1 analogs on ingestive behavior, cravings and craving control, and differential effects in the weight loss and weight maintenance phase. The measurement tools used for each outcome were also studied.

## Results

In order to shed light on the suggested relationships between GLP-1 analogs, ingestive behavior, and food preference, this review primarily focuses on studies which treated adults with obesity and/or type 2 diabetes with semaglutide and liraglutide, and which recorded measurements of appetitive behavior, consummatory behavior, and food preferences.

### Changes in ingestive behavior after treatment with GLP-1 analogs pharmacotherapy

Patients who used the GLP-1 receptor agonist exenatide displayed decreased neuronal responses to pictures of food measured by functional magnetic resonance imaging (fMRI) in parts of the brain that relate to appetite and reward (insula, amygdala, putamen, and orbitofrontal cortex) [36]. The glucagon-like peptide 1 receptor (GLP-1R) analog semaglutide, a once-weekly injectable medication for type 2 diabetes mellitus and obesity [37], targets the circumventricular organs rather than permeating the blood-brain barrier. By binding to GLP-1R in the subcortical areas of the brain, semaglutide has been suggested to potentially affect taste preferences and food intake behaviors via interaction with proopiomelanocortin neurons and cocaine- and amphetamine-regulated transcripts [38].

Patients treated with semaglutide undergo an initial phase of weight loss for 12–18 months followed by a weight stabilization phase [39]. During the initial phase, patients are more likely to experience adverse gastrointestinal side effects such as nausea, vomiting diarrhea, and constipation [40]. However, these effects can be attenuated by starting with lower doses of the medication and gradually titrating to the recommended dose [41]. Semaglutide resulted in reduction of body weight of up to 15% over 12–18 months in adults and adolescents with obesity [40]. The STEP 5 study evaluated semaglutide over 2 years which captured both the initial weight loss and weight-loss maintenance phases [42]. STEP 5 used verbal reports in the form of the Control of Eating Questionnaire to measure changes in appetite compared to a placebo. The group receiving semaglutide verbally reported lower cravings for dairy and starchy foods and less desire to eat salty or spicy foods, along with less difficulty controlling eating and resisting cravings. The initial changes seen during the weight loss phase were attenuated during the weight-loss maintenance phase [42], suggesting that once the patients achieve a new homeostatic fat mass, the biological drive to lose weight diminishes. Without the biological drive to achieve a lower body fat mass, there appears to be no physiological demand for changes to appetitive behaviors.

During the weight loss phase of semaglutide treatment, energy intake substantially decreased [43–47]. Decreased meal size and decreased preference for energy-dense foods are thought to contribute to the reduced energy intake associated with GLP-1R analogs [45] (Table 1). The macronutrient profile of foods selected by individuals taking semaglutide may change as shown in a randomized trial by Blundell et al. [45]. While in the weight loss phase, patients had decreased preference for high-fat, non-sweet foods as directly measured by consumption of an evening *ad libitum* snack box [45]. Using similar methods, a trial including 15 patients with type 2 diabetes mellitus (T2D) treated with oral semaglutide conducted by Gibbons et al. verbally reported a decreased preference for both high-fat and sweet foods from the snack box [48]. Measures of food preference were determined using data from verbal reports. Contrary to these findings, a cross-over study conducted in 1998 by Näslund et al. found no significant differences in the macronutrient profiles of foods selected by the group administered GLP-1 analog as compared to placebo [43]. Ingestive behavior using direct and objective measures not contingent on verbal reporting has not yet been reported for patients in the weight-loss maintenance phase, which raises the question whether the patients may return to baseline (pre-intervention) food choices.

**Table 1:** Studies investigating the role of GLP-1 analogs in body weight and ingestive behavior.

Author	Study type	Location	Population	GLP-1R Agonist	Methodology	Key findings
Näslund et al. [43]	Clinical trial	Stockholm, Sweden	Adults with obesity (BMI > 30 kg/m^2^)	GLP-1 infusion	Intravenous GLP-1 or saline was infused into 6 participants at the start of a controlled test meal along with 1.5 g acetaminophen. Blood sample analysis of acetaminophen, glucose, insulin, glucagon, and C-peptide was performed along with hunger, food choice, and fullness assessments.	Compared to control groups, GLP-1 group experienced reduced hunger, consumption and cravings as well as reduced rates of gastric emptying and postprandial blood glucose concentrations. GLP-1 infusion was not associated with change in energy consumed, rate of eating or plasma concentrations of insulin, glucagon, or C-peptide.
Näslund et al. [72]	Clinical trial	Stockholm, Sweden	Healthy men or post-menopausal women aged 18–65 with obesity	Recombiant GLP-1 (rGLP-1)	Prospective randomized, double-blind, placebo-controlled, cross-over study. rGLP-1 was administered 30 min before meals, four times daily, for five days.	rGLP-1-treated patients showed 15% reduction in mean food intake per meal, weight loss, and reduced gastric emptying.
Van Can et al. [44]	Randomized controlled trial	Maastricht, Netherlands	Adults aged 18–75 with BMI 30–40 kg/m^2^, stable body weight, and fasting blood glucose <7.0 mmol l^−1^	Liraglutide	Double-blind, incomplete crossover trial randomizing participants to two of three treatments: liraglutide 1.8 mg, 3.0 mg, or placebo. Energy expenditure, substrate oxidation, gastric emptying, glycemic parameters, appetite, and ad libitum energy intake as assessed by consumption of a lunch meal were assessed	Liraglutide doses increased satiety and fullness following meals. Hunger, prospective food consumption, and energy intake were decreased. Liraglutide-treated participants demonstrated a relative shift toward increased fat and reduced carbohydrate oxidation
Blundell et al. [45]	Clinical trial	Leeds, United Kingdom	Adults with obesity	Semaglutide	Randomized, double-blind, placebo-controlled, two-period crossover trial which assessed energy intake across lunch and evening meals, appetite ratings, thirst, nausea and well-being, control of eating, food preference, resting metabolic rate, body weight, and body composition.	24% energy reduction improved fasting appetite suppression, and lower preference for high-fat foods were reported with semaglutide treatment.
Kadouh et al. [54]	Randomized controlled trial	Rochester, Minnesota, USA	Overweight adults (BMI ≥ 27 kg/m^2^) and adults with obesity (BMI > 30 kg/m^2^), aged 18–65, local to Rochester, Minnesota	Liraglutide	After receiving standardized counseling on weight management, 40 participants began a 16-week trial of once-daily liraglutide or placebo.	Significant reduction in energy consumption, motivation to consume sweet, salty, savory, or fatty foods, as well as increases in postprandial GLP-1 levels and feelings of fullness were observed in the liraglutide group compared to placebo. The liraglutide group saw decreases in total body fat and increase in lean muscle mass.
Tronieri et al. [46]	Randomized controlled trial	Philadelphia, Pennsylvania, USA	Adults aged 21–70 years with BMI 30–55 kg/m^2^ and no serious medical or psychological conditions	Liraglutide	Appetitive changes were evaluated by randomizing participants to intensive behavioral therapy (IBT) alone, IBT with liraglutide, or IBT-liraglutide and a meal replacement diet. Hunger and food preferences were measured.	IBT-liraglutide participants experienced considerable weight loss, hunger reduction, and fullness increase
Friedrichsen et al. [47]	Randomized controlled trial	Berlin, Germany	Adults aged 18–65 with obesity (BMI 30.0–45.0 kg/m^2^)	Semaglutide	Double-blind, parallel-group trial. Gastric emptying, participant-reported appetite ratings, and energy intake measured by *ad libitum* lunch consumption were measured.	Energy intake was 35% lower with semaglutide. Hunger and prospective food consumption and increased fullness and satiety were reported. The CoEQ indicated improved eating control and fewer food cravings.
Gibbons et al. [48]	Randomized controlled trial	Leeds, United Kingdom	Adults aged 18–75 with type 2 diabetes for at least 90 days, HbA1c 6.0–9.0%, and body mass index (BMI) 20–38 kg/m^2^ who were treated with diet and exercise and/or stable dose of metformin for over 40 days	Semaglutide	Randomized, double-blind, placebo-controlled, two-period cross-over trial which measured energy intake determined by an *ad libitum* lunch and appetite ratings.	Energy intake following a standard breakfast was significantly lower in semaglutide-treated patients. Fewer food cravings and improved eating control were reported with semaglutide.
Wharton et al. [55]	Randomized controlled trial	Multi-city	Adults with a BMI ≥ 27 kg/m^2^ (with ≥weight-related comorbidity) or ≥30 kg/m^2^ without diabetes	Semaglutide	Data was pooled to analyze weight loss in the presence or absence of gastrointestinal adverse events (GI AEs)	GI AEs including nausea, diarrhea, vomiting, and constipation were more common with semaglutide than with placebo. Most GI AEs were non-serous and mild-to-moderate. Mean weight loss with semaglutide 2.4 mg was similar to that of participants without GI AEs than with.
Wharton et al. [42]	Randomized controlled trial	Multi-city	Adults with overweight or obesity	Semaglutide	Participants were randomized to semaglutide 2.4 mg or placebo, with lifestyle modification for 104 weeks. CoEQ was administered throughout in a sub-group of participants.	Participants administered semaglutide and who completed the CoEQ reported greater body weight reduction and improvements in craving domain scores. Cravings were significantly reduced with semaglutide.
Cawthon et al. [56]	Animal study	Florida, USA	Rats	Semaglutide	Rats received daily injections of a vehicle control or semaglutide titrated over time to maintenance dose	Rats treated with semaglutide reduced chow intake and body weight during dose escalation and maintenance. Energy intake as measured by chow consumption between semaglutide- and vehicle-treated rats were similar.

*CoEQ* Control of Eating Questionnaire, *BMI* body mass index, *GI AEs* gastrointestinal adverse events, *rGLP-1* recombinant GLP-1.

Liraglutide is a once-daily injectable GLP-1 receptor agonist also prescribed for the treatment of type-2 diabetes mellitus and obesity. Liraglutide initially reduces hunger and increases satiety [49]. Liraglutide similarly acts on GLP-1 receptors in the subcortical region of the brain and in the arcuate nucleus. Liraglutide also directly stimulates proopiomelanocortin neurons, and this has been postulated to result in reduced hunger and appetitive behavior [50, 51].

Liraglutide’s effect on slowing gastric emptying has been suggested as an explanation for higher satiety levels and decreased appetitive behavior, albeit the mechanism of this hypothesis has not been demonstrated [44]. Gastrointestinal side effects such as nausea were initially associated with weight loss in the phase 2 studies with liraglutide [52], but this observation was never confirmed, with all subsequent liraglutide studies showing nausea is not associated with weight loss [53].

### Effects on food cravings

Five previous studies used verbal reports to show decreased intensity of food cravings as well as a change in the types of foods they crave after GLP-1 analog treatment [43, 45, 47, 48, 54]. Using the Leeds Food Preference Task, the Blundell group found participants taking semaglutide craved a smaller number of foods, with a decrease in the implicit preference for high-fat, non-sweet foods in particular [45]. Gibbons also found semaglutide decreased preference of high-fat, non-sweet foods [48]. Using the forced choice list method, Näslund found GLP-1 analogs reduced craving for foods [43]. Furthermore, Friedrichsen using verbal reports showed the semaglutide group craved sweet, savory, and dairy-containing foods significantly less than the placebo group after 20 weeks of treatment and while in the weight loss phase [47]. Finally, Kadouh, using verbal reports also showed GLP-1R analogs lower preference for sweet, salty, fatty, and savory foods [54]. Ingestive behavior using verbal reports has not yet been reported for patients in the weight-loss maintenance phase, which raises the question again whether the patients may return to baseline (pre-intervention) food choices.

### Effects on control of eating

Verbal reports suggest that semaglutide improves short-term control of eating through decreased feelings of hunger associated with increased fullness and decreased severity of food within the first 12–24 weeks over 104-weeks [42]. The effect attenuated between 52 weeks and 104 weeks, but control of eating was still better than baseline.

### Differential effects during the weight loss phase and the weight maintenance phase

Semaglutide has a dose-escalating protocol to achieve a chronic maintenance dose. This is done to minimize side effects of the medication like nausea and malaise [55]. Most human studies on ingestive behavior have only focused on the weight loss phase with one exception of a 104-week study which also used verbal reports to describe appetite [42].

Cawthon et al. compared the metabolic, behavioral, and neural consequences of acute and chronic semaglutide administration in rats [56]. They confirmed the reduction in energy intake and loss of fat mass while maintaining lean mass in rats. Ingestive behavior during the weight loss and weight-loss maintenance phase over the span of 42 days was directly measured and showed that meal size and rate of eating had been the main modulators of semaglutide-induced changes in food intake.

In acute dosing protocols in rodents, where GLP-1R analogs were not dose titrated, as is the convention in humans, the drug reduces reward pathways that reinforce sucrose ingestion, thereby decreasing the amount of high-sugar-containing food that the rats consumed before they stopped ingesting calories [56–65]. When semaglutide was dose-titrated, like what typically happens in humans, the rats reached the weight-stable phase after approximately 10 days [56]. In the weight-stable phase, ingestion of low to mid-range sucrose-containing liquids was increased [56]. Rats consumed more than 2.5 times as much of low to mid sucrose-containing liquids as compared to control groups. Cawthon et al. identify several factors that could possibly explain this phenomenon, including the energy density of the stimuli, the stimuli’s texture (liquid vs. solid), the effects of GLP-1 analogs on taste responses, or changes in neurohormonal responses to the stimuli [56]. In this study, energy density was measured as the amount of energy or calories in a particular weight of food (kcal/g). Foods with lower energy density provide fewer calories per gram than foods with higher energy density which typically contain more fat, refined carbohydrates, and less fiber [66]. A deeper understanding of this and other consequences of chronic dosing schedules of GLP-1R agonizts will provide a more complete understanding of the long-term limitations and benefits of the medication, especially as it pertains to humans.

#### Measurement tools

##### Tools to measure ingestive behaviors

From its initial development by Kissilef et al. in 1980, the Universal Eating Monitor (UEM) provided the novel ability to test rates of consumption for solid and liquid foods, thereby considering consistency as a satiating influence [7, 67]. The UEM consists of a plate resting upon a scale which is connected to a computer. The scale tracks the weight of the food, transmitting the information to a computer very frequently throughout the process [5]. This allows for almost continuous monitoring of ingestive behavior, tracking changes in consumption speed throughout the meal, and provides much more information than simply weighing the meal before and after eating occurs [48]. Since its inception, the UEM has been adapted for broader human or animal studies to assess food intake patterns related to GLP-1 analog treatment. Näslund et al. used the tool in 1998 to measure total consumption, duration, eating rate, and relative rate of consumption in humans, which they defined as “food intake during the first half of the meal minus food intake during the second half of the meal divided by total food intake” [43].

Another tool for measuring ingestive behavior is the drinkometer [68]. Originally used to study ingestive behavior after bariatric surgery in humans, the device uses a liquid meal and tracks the volume consumed, length of consumption, number of sucks, and burst (group of sucks) size while the participant is drinking from the device [69].

Most of the human studies done to date relied on verbal reports and used a 100 mm visual analog scale (VAS) to assess satiety and fullness [43, 45–47, 54]. This consists of a 100 mm scale where participants mark their answers in response to questions such as “*How full do you feel*?” and “*How much do you think you can eat*?” [54]. Although the VAS is less expensive and more readily available for assessing a participant’s feelings of fullness compared to more direct measurement tools such as UEM and the drinkometer, the direct measures of behavior can circumvent the limitations and biases induced by verbal reporting.

##### Measures of diet composition after use of GLP-1 analogs

Gibbons et al. directly measured behavior by weighing food [48]. Participants with T2D received a snack box containing 100 grams of each of the following four types of food: high-fat, sweet; low-fat, sweet; high-fat, non-sweet; low-fat, non-sweet. The snack boxes were weighed before and after consumption to determine the composition of the food consumed by the participants. The study showed a decreased preference for both high-fat and sweet foods after treatment with GLP-1 analog [48]. The mean energy intake for high-fat sweet foods was 38.8% lower in the semaglutide-treated group than in the placebo group. Overall high-fat food intake was 40.8% lower in the oral semaglutide group when compared to the placebo group. Both variables represented statistically significant differences.

##### Measures of food preference

Blundell et al. used verbal reports and the Leeds Food Preference Task (LFPT) to examine changes in the choice of diet composition of adult humans with obesity and without diabetes [45]. The authors measured both explicit liking and implicit wanting of certain types of food using photos of different foods that were either sweet or savory or high-fat or low-fat. In the LFPT, explicit liking is measured by having participants rate how pleasant the food in each photo is to them. To study implicit wanting, the authors employed forced choice. Participants were forced to choose between sets of photos from the four categories based on which they would want the most, with shorter response times corresponding to a stronger desire for the chosen photo. Similarly, Näslund et al. studied food preference of adult humans with obesity through a forced choice approach [43]. Participants had to choose from different groups of foods– high protein, high-fat, high-carbohydrate, and low energy– to determine which they preferred [43, 45]. In their study, Friedrichsen used the Control of Eating Questionnaire (COEQ) to assess different preferences of adult humans with obesity [47]. Finally, Kadouh et al. used a 100 mm VAS to measure preference for fatty, salty, sweet, and savory foods [54]. In a randomized controlled trial, Griffioen-Roose et al. compared the LFPT with other measurements of desire for different foods and sensory-specific satiety [70]. They use three measurements– LFPT, *ad libitum* intake (measured by giving participants bowls of different types of food and weighing the remaining food in each bowl), and willingness to work, where participants play a computer game and try to get enough points to win food (measured by mouse clicks until the game is stopped). The authors found no difference in results based on the measurement procedure used. LFPT/forced choice and COEQ all rely on verbal reporting, but the willingness to work and *ad libitum* intake are direct measures of behavior. It was reassuring that no major differences were found between the verbal report measures and the direct measures of behavior [45, 70, 71].

##### Measures of eating control

Along with preference, the COEQ was used by several researchers [42, 45, 47, 48]. These verbal reports showed that after GLP-1 analog treatment, in all four studies, control of eating became easier.

## Discussion

Recent developments have introduced GLP-1 analogs as a highly viable option for the treatment of obesity, representing the newest generation of therapeutic models for weight management. Several studies have aimed to shed light on the mechanisms driving pharmacotherapy-induced weight loss by investigating their impact on ingestive behavior. Altered food preferences, decreased food cravings, and reduced food intake may contribute to long-term weight loss.

However, since the approval of GLP-1R analogs for the treatment of obesity, surprisingly few studies have been done to understand ingestive behavior. Studies are further complicated by the distinct and potentially unrelated natures of food intake (i.e., the consumption of food as a result of behaviors) and food preference (i.e., a subject’s food choices dependent on their preferences and personal enjoyment) as separate and potentially unrelated variables. Very little is known about appetitive behavior and almost nothing is known about consummatory behavior, especially in the weight maintenance phase. The studies done thus far mostly focused on the weight loss phase and predominantly used verbal reporting to understand appetite, cravings, and portion control. Moreover, the tools leveraged by these studies analyze multiple variables of food preference, which is undoubtedly imperative in understanding how they interplay to impact food behaviors. However, it is ultimately crucial to investigate objective measures of food intake to better clarify the effect of anti-obesity medications. Direct measurement of behavior is possible using new equipment which has been validated in the study of gastric bypass surgery. This may provide more reliable results.

While a variety of tools have been repurposed and refined to assess food behaviors, each measurement technique has accompanying limitations to account for. For instance, the UEM provides opportunities for dynamic consumption pattern measurements, but restricts investigators to a single set of food items under a laboratory setting. This not only limits the conditions under which food behavior can be explored, but also introduces a potentially confounding variable of the discomfort which may come with participants eating in a laboratory. The Drinkometer assesses multiple crucial variables of ingestive behavior, but the short duration between sucks and bursts limits opportunities to extract detailed analyses of interval measurements. Moreover, the relatively high expenses associated with the tool present limitations to research. Contrarily, the VAS is less expensive and accessible for most researchers. However, responses from study participants are subject to individual interpretation of scale ratings and introduce an element of subjectivity to acquired results. The LFPT is uniquely able to simultaneously measure explicit liking and implicit wanting of participants, but does not account for variation in food preferences which may present across different times in a day. While the CoEQ can measure food cravings over longer periods of time and is not limited to static points in a given day, this method does not allow for an objective, actual measurement of intake across participants. It is prudent to consider each of these strengths and limitations and accordingly design effective and optimal future investigations (Table 2).

**Table 2:** Methods and tools for assessment of food intake behavior in humans.

Tool	Food Behavior Assessed	Mechanism	Strengths	Limitations
Universal Eating Monitor (UEM)	Food intake	Food is continuously weighed on a scale throughout meal consumption	Dynamic consumption patterns are explored instead of static measurements before and after a meal	Measurements are restricted to one set of food items under one condition; eating in a laboratory setting which could be unusual for participants [7]
Drinkometer	Food intake	A liquid meal is consumed through a straw	Ingestive patterns including volume consumed, sucks, and bursts are tracked	Interval measurements between sucks and bursts are limited, an expensive tool [69]
Visual analog scale (VAS)	Food preference	Participants mark responses to hunger and consumption related questions on a 100 mm scale	Less expensive and readily available	Ratings on the scale are subjective and interpretations can vary between participants [73]
Leeds Food Preference Task (LFPT)	Food preference	Participants choose between various categories of food to rate their desirability	Explicit liking and implicit wanting are simultaneously measured	May not account for food preferences across times of day [74]
Control of Eating Questionnaire	Food preference/eating control	Participants respond to twenty-one items over six sections based on their experience over the past seven days	Specific food cravings can be identified over periods of time	Actual intake across participants is not measured [75]

The treatment options for obesity are rapidly improving, and more and better medications will become available. By understanding the behavioral mechanisms which explain how anti-obesity medications impact ingestive behavior, researchers can potentially refine treatment strategies and tailor interventions to individual patients. This may enhance the overall success of obesity management with these medications. With direct measures of ingestive behavior in animals now suggesting that low to mid-range sucrose intake may increase after semaglutide treatment, it is imperative to study ingestive behavior in humans using direct measures. The focus should also be on the weight maintenance phase, because the absence of such information risks the propagation of misinformation and the harboring of unrealistic expectations by both clinicians and patients, which can influence patients’ and clinicians’ willingness to use anti-obesity medications in the future.
